# Repeated buffered acidic saline infusion in the human masseter muscle as a putative experimental pain model

**DOI:** 10.1038/s41598-019-51670-3

**Published:** 2019-10-29

**Authors:** Sofia Louca Jounger, Niklas Eriksson, Helena Lindskog, Anna Oscarsson, Vivian Simonsson, Malin Ernberg, Nikolaos Christidis

**Affiliations:** 1 Division of Oral Diagnostics and Rehabilitation, Department of Dental Medicine, Karolinska Institutet, SE-141 04 Huddinge, Sweden; 2 Scandinavian Center for Orofacial Neurosciences (SCON), Huddinge, Sweden

**Keywords:** Medical research, Signs and symptoms

## Abstract

This study investigated if repeated buffered acidic saline infusions into the masseter muscles induced muscle pain and mechanical sensitization. Fourteen healthy men participated in this double-blind, randomized, and placebo-controlled study. Two repeated infusions (day 1 and 3) were given in the masseter muscles with either a buffered acidic saline solution (pH 5.2) or an isotonic saline solution (pH 6) as control. After 10 days of wash-out, the experiment was repeated with the other substance. Pressure pain thresholds (PPT), pain intensity, maximum unassisted mouth opening (MUO), and pain drawings were assessed before, directly following, and after each infusion at 5, 15, and 30 min and on day 4 and 7. Fatigue and pain intensity were assessed after a one-minute chewing test 30 min after infusions and day 4 and 7. Acidic saline induced higher pain intensity than control day 3 up to 5 min after infusions, but did not affect PPT. The chewing test did not evoke higher fatigue during chewing or MUO or after acidic saline infusion compared to control. Repeated acidic saline infusions in the masseter muscles induced a short-lasting muscle pain without mechanical hyperalgesia or functional pain. Hence, this model might not be superior to already existing experimental muscle pain models.

## Introduction

Chronic musculoskeletal pain disorders affect nearly one-third of the world’s population^1,2^ and are associated with significant individual disability and suffering. In the orofacial region about 7–11% of the population suffer from chronic pain, making it one of the most frequent locations for chronic pains^3,4^. In similarity to many other musculoskeletal pain disorders orofacial pain is more common in women than in men^5^. Pain conditions in the orofacial region including the temporomandibular joints (TMJ), the masticatory muscles, and associated structures, such as surrounding bones and immediate tissue components are all embraced under the term temporomandibular disorders (TMD). The pain overlaying the masticatory muscles (TMD myalgia; M-TMD) and/or the TMJ is usually described as a dull steady pain, and is commonly referred to other craniofacial structures, such as the teeth. M-TMD is frequently accompanied by restricted jaw opening, pain upon chewing, muscle soreness, headache, and also by anxiety and depression^6,7^.

The etiology and pathogenesis of TMD and its higher prevalence in women is poorly understood^8^. In order to improve our knowledge about chronic M-TMD, diagnostics, functional impairments and therapeutics, human experimental pain models that mimic these pain conditions are essential. Such pain models are essential for bridging the gap between basic science and clinical application. Thus, standardized, easy, and safe human experimental pain models are needed in order to better understand the mechanisms behind M-TMD, any possible chewing and biting deficiencies caused by M-TMD, and also to be able to improve diagnostics and treatment.

Previous studies have shown that there is a positive correlation between development of muscle pain and local acidity in the muscle^9–11^, indicating that an experimental model with acidic saline injections would be useful. A previous study has shown that two repeated acidic saline (pH 4) injections into rats gastrocnemius muscle, two to five days apart caused a mechanical hyperalgesia for 30 days. Furthermore, these repeated intramuscular injections showed an effect on the contralateral side^12^. However, the long-lasting mechanical hyperalgesia produced seems to depend on the dosage, the duration of infusion, and the pH of the solution, whereas, the lower pH (pH 4) the greater hyperalgesia^12^. Also, the interval between injections seem to be of great importance since by increasing the interval from two or five days to 10 days prohibited the development of long-lasting mechanical hyperalgesia^12^. With this in mind, one can assume that there is a critical time-frame in which the repeated trauma of the muscle tissue post-infusion results in an exaggerated, more long-lasting hyperalgesia.

It is at present unclear whether the acidic saline model evoke similar effects in orofacial muscles as in limb muscle in rodents, since previous studies show conflicting results. In one study, repeated acidic saline (pH 4) injection into rat masseter muscle two days apart caused long-lasting mechanical allodynia^12^, while another study with similar methodology failed to induce mechanical allodynia^13^.

A few previous human studies have used intramuscular infusions of acidic saline to induce pain of muscular origin. We have shown that two repeated injections into the human masseter muscle, two days apart, with an un-buffered acidic saline solution with a pH of 3.3, resulted in mild short-lasting pain, but no mechanical allodynia^14,15^. Another study from our group, using two repeated infusions with un-buffered acidic saline with a pH of 3.3 in the human masseter muscle did not find any increase of algesic or metabolic substances (serotonin, glutamate, glucose, lactate, pyruvate)^14^. These results combined could indicate that the Sluka model^12^ cannot be directly translated into human jaw muscles. However, in another study a single infusion of a buffered acidic saline solution with pH 5.2 into a pain-free human tibialis muscle, resulted in mild to moderate muscle pain intensity, pain referral, and also mechanical allodynia lasting 20 min^9^. Hence, intramuscular injections with a buffered acidic saline solution (pH 5.2) may induce muscle pain more effectively than an un-buffered solution with even lower pH, since it take longer time for the tissue to regain a normal pH.

With this in mind, we hypothesized that it might be necessary to use a buffered acidic saline solution to induce both muscle pain and mechanical allodynia, and thus to better mimic the clinical M-TMD condition. Therefore, the aim of this study was to investigate if two repeated infusions of a buffered acidic saline solution into the human masseter muscles would induce muscle pain and mechanical sensitization.

## Results

All the participants participated on both day 1 and 3 at both sessions. However, one participant dropped-out after the second infusion of the second experiment, since he received braces and experienced a high intensity of pain spread all over the face. This was reported as “missing data”.

### Changes in pain characteristics

#### Pain intensity during infusions

**Figure 1:**
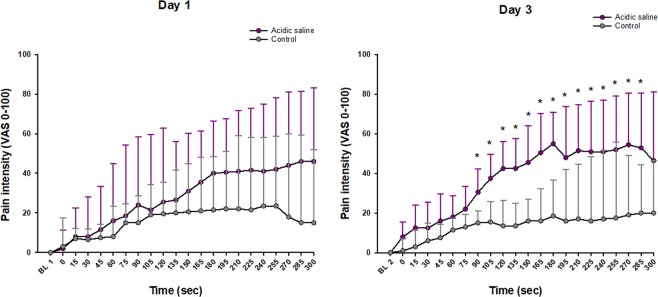
Graph showing the median (SEM) pain intensity assessed with a 0–100 mm Visual analogic scale (VAS) before and at various time points during two repeated infusions of acidic saline and isotonic saline (control) into the masseter muscle of 14 healthy men at day 1 and day 3. *Significant difference between acidic saline and control, *P* < 0.001.

The pain intensity after infusions is shown in Fig. 1. None of the participants reported any pain at baseline. The buffered acidic saline solution induced a significantly higher pain intensity compared to control during the infusions on day 3 (Wilcoxon test; *P* < 0.001) starting at time point 90 sec up to 285 sec. The pain intensity changed significantly over time after acidic saline infusion (Freidman test; day 1; *P* = 0.040, day 3; *P* = 0.025). The posthoc test showed that it was significantly increased compared to baseline from 90 sec and onwards at day 1 (Tukey test; *P* = 0.040) and from 105 sec and onwards at day 3 (Tukey test; *P* = 0.025). On the control side, the pain intensity changed over time (Freidman test; day 1: *P* = 0.029, day 3; *P* = 0.023). The posthoc test showed that it was significantly increased compared to baseline from 105 sec at day 1 (Tukey test; *P* = 0.029) and from 165 sec day 3 (Tukey test; *P* = 0.023).

#### Pain intensity after infusions

There was a significantly higher pain intensity at rest (NRS) at the time point 5 min on the acidic saline side compared to control on day 3, but not on any other time points or side, as shown in Table 1. There were significant differences in pain intensity over time on the acidic saline side (Friedman test; *P* < 0.001) and on the control side (Friedman test; *P* = 0.001). The post hoc test showed a significant difference at day 1, 3, and 7 between the time point 5 min compared to baseline (Tukey test; *P’s* < 0.032) on the acidic saline side but no significant difference on the control side (Tukey test; *P* = 0.074).

**Table 1:** The median (IQR) values are shown for the maximal unassisted mouth opening (MUO), pain intensity at rest and pain area before (BL) and 5, 15, and 30 min as well as at day 4 and day 7 after two repeated infusions of buffered acidic saline or isotonic saline (control) into the masseter muscle of 14 healthy men at day 1 and day 3.

	Acidic saline	Isotonic saline	*P*-value
**MUO (mm)**			
**DAY 1**			
BL 1	55.0 (2.8)	55.0 (4.5)	0.250
5	54.5 (7.8)	55.0 (6.3)	0.383
15	54.5 (8.8)	55.5 (6.3)	0.232
30	55.5 (5.5)	55.0 (5.5)	0.638
**DAY 3**			
BL 2	55.0 (5.0)	55.5 (6.3)	0.677
5	53.5 (11.8)	54.5 (5.3)	0.175
15	54.0 (9.3)	54.0 (5.5)	0.733
30	54.5 (7.0)	55.5 (3.5)	0.151
**DAY 4**	56.0 (5.5)	55.0 (7.0)	0.652
**DAY 7**	54.5 (4.8)	55.5 (8.0)	0.839
**PAIN INTENSITY at rest (0–10 NRS)**			
**DAY 1**			
5	1.5 (4.3)	1.0 (2.0)	0.078
15	0.5 (3.0)	0.5 (1.0)	0.109
30	0.0 (1.0)	0.0 (0.0)	0.375
**DAY 3**			
5	3.0 (4.5)	1.0 (3.0)	0.005*
15	1.0 (2.3)	0.0 (1.3)	0.063
30	0.0 (1.5)	0.0 (0.3)	0.250
**DAY 4**	0.0 (0.0)	0.0 (0.0)	0.335
**DAY 7**	0.0 (0.0)	0.0 (0.0)	Na
**PAIN AREA (au)**			
**DAY 1**			
5	26.5 (96.3)	34.0 (57.3)	0.695
15	2.0 (22.8)	7.0 (24.0)	0.734
30	0.0 (5.3)	0.0 (3.3)	0.563
**DAY 3**			
5	16.0 (842.0)	6.5 (17.5)	0.064
15	0.0 (12.3)	0.0 (12.8)	0.747
30	0.0 (0.0)	0.0 (0.0)	Na
**DAY 4**	0.0 (0.0)	0.0 (0.0)	Na
**DAY 7**	0.0 (0.0)	0.0 (9.5)	1.0

*Significant difference (Wilcoxon test; *P* = 0.005) Na = not applicable.

#### Pain spread

There were no significant difference in pain spread (au) between the substances at any of the time points (Wilcoxon test; *P* > 0.05), as shown in Table 1. There was a significant difference in pain spread over time on the acidic saline side (Friedman test; *P* = 0.006), but the post hoc test could not detect any time points that differed from baseline (Tukey; *P* = 0.303). There was no significant difference in pain spread on the control side (Friedman test; *P* = 0.468).

#### Pain duration

There was no significant difference in pain duration (min) after infusions with acidic saline or control at day 1 and day 3, or between the two substances at day 1 and day 3 (Table 2).

**Table 2:** The median (IQR) values are shown for the pain duration (min), pain evoked by a 1-minute chewing test (0–10 NRS), fatigue (Borg 6–20), and pain quality (MPQ PRI) before (BL) and directly after (day 1 and 3) as well as at day 4 and day 7 after two repeated infusions of buffered acidic saline or isotonic saline (control) into the masseter muscle of 14 healthy men at day 1 and day 3.

	Acidic saline	Isotonic saline	*P*-value
**PAIN DURATION**			
DAY 1	8.0 (3.0)	7.0 (1.0)	0.125
DAY 3	8.0 (8.0)	6.0 (2.0)	0.063
	*P* = *0.219*	*P* = *0.188*	
**CHEWING PAIN**			
BL 1	0 (0.0)	0 (0.0)	Na
DAY 1	0.0 (0.0)	0.0 (0.0)	Na
BL 2	0.0 (0.0)	0.0 (0.0)	Na
DAY 3	0.0 (1.0)	0.0 (1.0)	0.317
DAY 4	0.0 (0.0)	0.0 (0.0)	Na
DAY 7	0.0 (0.0)	0.0 (0.0)	Na
**FATIGUE**			
BL 1	6.0 (0.0)	6.0 (0.0)	Na
DAY 1	8.5 (4.5)	7.0 (4.3)	0.243
BL 2	6.0 (0.0)	6.0 (0.0)	Na
DAY 3	9.0 (4.5)	6.0 (3.3)	0.133
DAY 4	6.0 (5.0)	6.0 (4.0)	0.870
DAY 7	6.0 (4.0)	6.0 (3.0)	0.669
**PAIN QUALITY (MPQ PRI)**			
DAY 1	14.5 (15)	8.0 (8.0)	0.048
DAY 3	15.5 (20.5)	8.5 (13.8)	0.836
	*P* = *0.641*	*P* = *0.542*	

Na = not applicable; MPQ = McGill Pain Questionnaire; PRI = Pain Rating Index.

#### Pain quality

There were no significant differences in PRI scores over time or between substances as shown in Table 2.

#### Chewing pain and fatigue

The pain evoked by the 1-minute chewing-test, did not differ significantly between the two substances (Table 2). There was a significant difference in chewing pain over time on the acidic saline side (Friedman test; *P* = 0.028), but the post hoc test could not identify which time points that differed (Tukey test; *P* = 0.069). There was no significant change over time at the control side (Friedman test; *P* = 0.078). Neither were there any differences in perceived fatigue between the two substances (Table 2). The perceived fatigue did not differ significantly over time on the acidic saline side (Friedman test; *P* = 0.069) or the control side (Friedman test; *P* = 0.275).

### Maximal unassisted mouth opening

There were no significant differences (Wilcoxon test; *P* > 0.05) in MUO between the substances at any time point (Table 1). Neither did MUO change over time on the acidic saline side (Friedman test; *P* = 0.101) or the control side (Friedman test; *P* = 0.335).

### Pressure pain threshold

**Figure 2:**
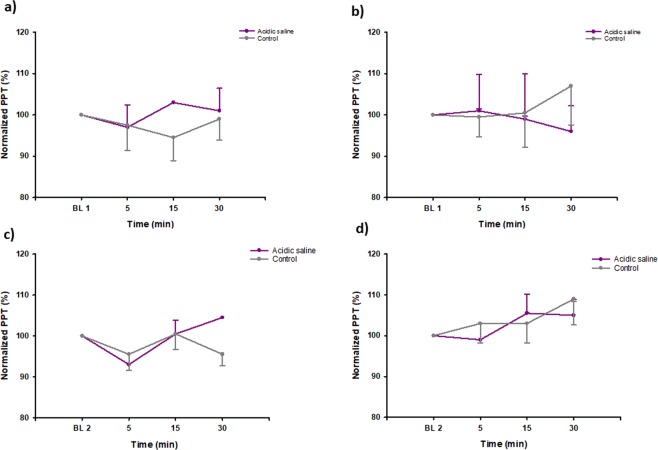
The figure illustrates the changes (%) of pressure pain threshold (PPT) at the masseter and temporalis muscles before (BL) and 5, 15 and 30 min after two repeated infusions of acidic saline and isotonic saline (control) into the masseter muscle on the ipsilateral side i.e. the side of the infusions of 14 healthy men at day 1 (D1) and day 3 (D3). The figures show the normalized PPT on the ipsilateral side in (**a**) the masseter muscle day 1, (**b**) the temporalis muscle day 1, (**c**) the masseter muscle day 3 and (**d**) the temporalis muscle day 3.

**Figure 3:**
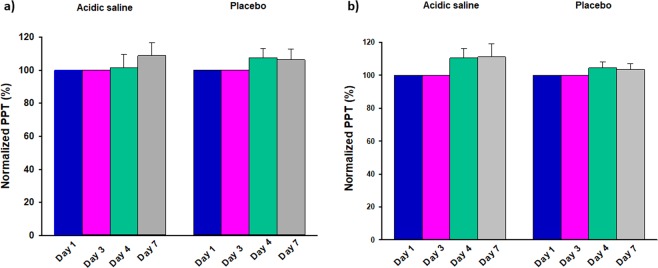
The figure illustrates the changes (%) from baseline of the pressure pain threshold (PPT) on the ipsilateral side in (**a**) the masseter and (**b**) the temporalis muscle after two repeated infusions two days apart of buffered acidic saline or isotonic saline (control) into the masseter muscle on the ipsilateral side i.e. the side of the infusions of 14 healthy men. PPTs were assessed directly after infusions on day 1 and 3 as well as on day 4 and 7. None of the substances induced any change in PPT at any time points.

PPT assessments over the masseter and temporalis muscles are shown in Figs 2 and 3. There were no significant changes with time, neither in the PPTs at the ipsilateral masseter (injection site), nor contralateral masseter muscle, or at any of the temporalis muscles.

There were further no significant differences between the two substances at any of the time points.

The two-way mixed-model ANOVA showed no significant time effect (*F* = 0.148; *P* = 0930), treatment effect (*F* = 1.995; *P* = 0.181) or interaction between time and treatment (*F* = 0.723; *P* = 0.544).

## Discussion

The main finding was that two repeated infusions of sterile buffered acidic saline (pH 5.2) two days apart into the masseter muscle resulted in pain of moderate intensity lasting approximately 10 min. The acidic saline induced pain intensity was shown to be significantly higher than the control substance during infusion as well as 5 min after on day 3. However, the model induced no detectable mechanical hyperalgesia or functional pain (during MUO and chewing) or affected the MUO in this relatively small sample size. Together this might indicate that this model is not superior to other human experimental pain models for chronic orofacial muscle pain, such as hypertonic saline or glutamate injections^16–19^.

While our earlier studies with repeated injections of an un-buffered acidic saline (pH 3.3) caused only mild (NRS 3) and short-lasting levels of pain^14,15,20^, the pain intensity in the present study was slightly higher (NRS 5) and differed from normal saline on day 3. However, the pain was still short-lasting. This is in agreement to other experimental pain models, for example those using injection or infusions of hypertonic saline or glutamate into the human masseter muscle causing short-lasting pain^16–18,21^ with an average peak-pain intensity around NRS 5–6^16,21^. This indicates that repeated infusions with buffered acidic saline evoke pain of similar intensity and duration as these other human experimental pain models for masseter myalgia. The higher pain intensities on day 3 is an interesting finding that could indicate that, even if we could not detect any sensitization to mechanical stimuli, sensory afferents may still have been sensitized. It has been speculated that the decrease in pH following infusions of acidic saline result in activation of chemosensitive nociceptors on primary afferent neurons. These primary afferent neurons can for instance be the transient receptor potential vanilloid 1 (TRPv1), and/or the acid-sensing ion channels 1 and/or 3 (ASIC1 and/or ASIC3)^9,10^. The channels opens when the pH is lowered. This local reaction can be observed as a response to inflammation or metabolic breakdown products^10^.

The intention of using un-buffered solutions in earlier studies was to emulate the original animal model by Sluka and co-workers as closely as possible^12^. This may account for the default in producing pain and mechanical hyperalgesia since an un-buffered saline solution could reach physiologic pH-levels quick compared to a buffered solution due to the buffering capacity of human muscle tissue. The longer the pH remains acidic in the muscle, the longer the ASIC3 channels are able to continue generating sustained currents, i.e. be activated, and hence hypothetically produce pain and mechanical hyperalgesia^10^. This could indicate that a buffered acidic saline solution would be superior to an un-buffered as a human pain model and was the reason for the use of a buffered solution in this study.

It has been shown, in a previous animal study that the lowest pH on average that could be gained after an acidic saline (pH 4) intramuscular injection into the gastrocnemius muscle was 6.5^12^. In another animal study where hyaluronic acid was injected subcutaneously into rat hind paw, the assessed intramuscular pH did not reach to levels under pH 5, and the pH was normalized after just 15 min^22^. Thus, it seems favorable to use a buffered acidic solution as compared to an un-buffered solution. This is further supported by three earlier studies^9,11,23^ where a buffered acidic solution (pH 5.2) was infused into both human tibialis anterior as well as forearm muscles caused mild to moderate pain, but also mechanical hyperalgesia, which contrasts our results. Perhaps the difference regarding mechanical hyperalgesia could be due to the different injection sites, i.e. jaw versus limb muscles, as discussed previously^14,15,20^. In histological comparisons it has been shown that there is a better capillary supply in the masseter muscle than in the tibialis anterior^24^. This could lead to a quicker clearance of acidic solution out of the masseter muscle, which in turn would lead to a lesser possibility to maintain a low pH. Furthermore, based on novel data there are some indications that there is a larger amount of putative nociceptive nerve fibers in the connective tissue of the masseter muscle than the tibialis anterior muscle^25^. Other explanations between limb and jaw muscles are different muscle size and motor functions. While leg movements mainly include dynamic contractions of limb muscles, chewing and tooth clenching include both dynamic and static contractions^24,25^. Taken together, it seems that jaw muscles respond in a different way than limb muscles regarding buffered acidic saline injections.

The original pain model developed by Sluka and co-workers^12^ is unique in inducing long-lasting mechanical hyperalgesia. A key element is also the spread of hyperalgesia to the contralateral side. Both these mechanisms reside in the central nervous system and are not dependent on continuous nociceptive input from the periphery^12^. In other words, the maintenance of muscle sensitization and pain spread are thought to be central phenomenon. Thus, the lack of mechanical sensitization and pain spread in this study indicate that no central sensitization occurred. On the other hand, earlier studies in the human masseter muscle^14,15,20^ indicate that other mechanisms also seem to participate in developing mechanical orofacial hyperalgesia, such as muscle fiber composition and capillary supply^15,24^. Consistent with this assumption, experimentally induced muscle pain in humans seem to result in different pain thresholds and characteristics for the muscles innervated by the trigeminal nerve than for muscles innervated by the cervical spinal nerves^26^.

Another aspect that can be addressed is the infusion rate, since it has been discussed as a factor affecting the outcome of the pain model. A previous study showed that infusion rates between five to 40 mL/h (mean rate 16 ± 3.7 mL/h) were needed to reach pain scores of 20 and higher on a VAS 0–100^11^. Another study reported that an infusion rate of 40 mL/h instead of 20 mL/h induced pain of higher intensity and lower PPT values^9^. Taken together, the participants in these two studies developed muscular pain of a dull-aching or stinging character, with a magnitude that showed a log-linear correlation in comparison to the flow rate. By increasing the infusion rate the pain intensity scores increase, since this results in a more effective lowering of the local pH and an increase in tissue volume in which the proton concentration exceeds the threshold to excite nociceptors^27^. In these studies^9,11^ injections were made into larger muscles compared to the masseter which makes it difficult to directly compare results.

A few limitations need to be addressed. The experiments were performed by two different examiners, which may have affected the results. However, both were trained thoroughly in the experimental protocol, including the DC/TMD examination (by a gold-standard examiner, ME, Malmö Center), and the procedure was performed in exactly the same manner. Also, both examiners were female to make sure that there would not be a difference in outcome between the examiners due to the gender aspect^28^. Another limitation is that only men were included. Previous studies have shown sex differences to algesic muscle injections^16,19^, so we do not know if the results would be the same in women. Future studies can be conducted to address this. Finally, the sample size in this study was smaller than in previous studies. It can therefore be argued that it was underpowered to detect mechanical hyperalgesia. Indeed, the study by Frey Law and co-workers that found a weak (10–15%) but significant reduction in PPTs of the tibialis muscle included a larger sample size^9^. On the other hand, an optimal experimental pain model should preferably have a strong effect size to minimize the number of volunteers. Nevertheless, future studies can be conducted with a larger sample size in order to address this before any definite conclusions can be drawn about the model.

## Conclusion

Repeated infusions with buffered acidic saline into the masseter muscle induced a short-lasting muscle pain, and no hyperalgesia or functional pain was detected with the current sample size. Hence, we suggest that this model might not be superior to already existing human experimental pain models for chronic orofacial muscle pain.

## Methods

The project was approved by the Regional Ethical Review Board in Stockholm, Sweden, (2014/466-31/4) and followed the guidelines according to the Declaration of Helsinki as well as Good Clinical Practice. The participants received both verbal and written information about the study, and gave their written informed consent.

### Participants

Fourteen healthy male participants with a mean (±SD) age of 25.4 (±3.6) years were included in this study. Inclusion criteria were: (**a)** age between 18–40 years, and (**b)** good general health. Exclusion criteria were: (**1)** self-reported muscular or joint pain in the orofacial region, (**2)** a diagnosis of TMD myalgia according to the Diagnostic Criteria for TMD (DC/TMD)^4^, (**3)** palpatory tenderness over the masseter muscles, (**4)** local skin infection at the region of the injection site, (**5)** systemic inflammatory disease, such as rheumatoid arthritis or fibromyalgia, (**6)** whiplash associated disorder, (**7)** neurological or psychiatric disorders, (**8)** neuropathic pain or pain of dental origin, (**9)** recurrent use of muscle relaxants, and (**10)** anti-inflammatory or analgesic medication 48 hours prior to the experiment.

According to the power analysis, inclusion of at least 13 participants would be sufficient to detect statistically a clinically significant difference (30%, SD 35%) of pain intensity between substances with a power of 80% and a significance level of p < 0.05.

### Experimental protocol

The study used a double-blind, placebo-controlled, and randomized cross-over design. The randomization of the order in which the infusions were given was performed by a computer (www.randomization.com), by a person not participating in the experiment (NCh). The right side of the masseter muscle was chosen for all the injections. The preparation and blinding of the syringes was done by either of two researchers not participating in the study (ME/NCh). Since isotonic saline and acidic saline have identical appearances, the participants and the investigators were completely blinded. The experiments were conducted in a quiet room with the participants seated in a conventional dental chair.

**Figure 4:**
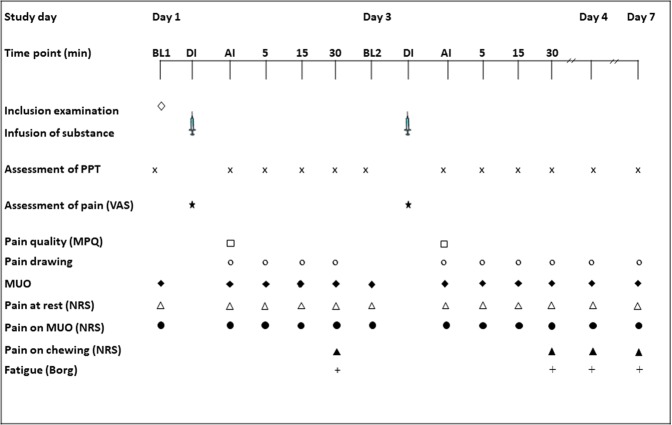
The experimental protocol show the time points for the inclusion examination and the various assessments after infusion of buffered acidic saline or normal saline into the masseter muscle of 14 healthy men. Pain was assessed (VAS) every 15 sec during the 5-min infusion. The pressure pain thresholds (PPTs), maximum unassisted mouth opening (MUO), and pain at rest and on MUO were assessed at baseline and directly after infusion and then every 5 min up to 30 min after infusion. Pain quality (McGill) was assessed directly after infusions, and a pain drawing completed directly after and then every 5 min up to 30 min after infusion. A 1-min chewing test was performed 30 min after infusion, and the pain on chewing (NRS) and fatigue (Borg) were assessed. DI = During infusion, AI = After infusion, VAS = 0–100 mm visual analogue scale, NRS = 0–10 numerical rating scale.

All subjects participated in two experiments, one with buffered acidic saline and one with normal isotonic saline as control. Both experiments consisted of four appointments. At the first visit (day 1) the participants were informed about the study protocol and a clinical examination according to DC/TMD Axis I^4^ was performed in order to assure that the participants fulfilled the inclusion and none of the exclusion criteria. Baseline measurements of pressure pain threshold (PPT) and maximum unassisted mouth opening (MUO) were recorded and the first infusion i.e. either buffered acidic saline or control solution was given. During the infusion pain intensity was repeatedly assessed. Directly after the infusion (AI) PPT, pain quality, pain spread, MUO, and pain on MUO were assessed. PPT, pain spread, MUO, and pain on MUO were then assessed at the time points 5, 15, and 30 min after the infusion. A chewing task was also performed at the time point 30 min and pain and fatigue in response to the task were recorded. At the second visit (day 3), the procedure was repeated in exactly the same manner, i.e. the same substance and muscle were used for infusion. The first two visits lasted for approximately 45 min. During the third and fourth appointment (day 4 and 7) a follow-up was performed including a clinical examination according to DC/TMD, assessments of pain characteristics (pain intensity and spread), PPT, MUO as well as the chewing task. Each follow-up appointment lasted for approximately 15–20 min. After 10 days of wash-out, the experiment was repeated in the same manner with infusion of the other substance into the masseter muscle on the same side (Fig. 4).

### Assessment of pain characteristics and fatigue

To assess the intensity of pain experienced by the participants during the infusion, 0–100 mm visual analogue scales (VAS) marked with the end-points “no pain” and “the worst imaginable pain experienced” were used. Pain intensity was assessed every 15 seconds until pain subsided with a maximum extend to 300 sec. The pain duration was recorded in minutes. Further, a 0–10 numeric rating scale (NRS) was used to assess pain intensity at rest and in response to MUO and chewing after the infusions.

To record the maximum spread (area) of the pain experienced, pain drawings of the lateral view of the head were used, and the pain area was assessed in arbitrary units (au)^15,29^.

For pain quality the participants were asked to complete the validated Swedish version of the McGill Pain Questionnaire (MPQ)^30^. The questionnaire contains 78 adjectives divided in 20 groups and can be used to describe different aspects of pain quality (sensory, affective and miscellaneous). Each group contains a varying number of adjectives that are ranked according to severity. The participants were instructed to select the adjectives that best described their own pain experience. The total score of the ranks was calculated for each participant as the pain rating index (PRI)^31^. Fatigue evoked by the chewing test was assessed using the Borg’s Rating of Perceived Exertion (RPE) Scale; 6–20^32^. The Borg’s RPE scale ranges from 6–20, where 6 equals “no exertion”, and 20 “maximum exertion”^32^.

### Maximum unassisted mouth opening

A metal ruler (Limit, Germany) was used to record the MUO according to the instructions in the DC/TMD protocol^4^. The recordings were done once and registered in millimeters (mm) from the center of the incisal edge of the central incisor of the upper jaw to the incisal edge of the corresponding tooth in the lower jaw.

### Assessment of pressure pain threshold (PPT)

An electronic algometer (Somedic Sales AB, Hörby, Sweden) was used to assess PPT bilaterally at the masseter and temporalis muscles. The participants were instructed to press a button as soon as the sensation of pressure changed into pain. The tip of the algometer was 1 cm in diameter and covered by a soft, rubber material. During the assessment, the algometer was held at a 90-degree angle to the skin-surface overlaying the most prominent part of the masseter muscle and the anterior part of the temporalis muscle. The increase in pressure was set to a rate of 50 kPa/s. At the two baseline sessions for the buffered acidic saline and control substance day 1 and on day 3 the recordings were assessed three times, whereas the rest of the recordings were done twice, all with an interval of 2 min in-between measurements. The mean value from these measurements were used in the statistical analysis.

### Infusions

The participants were asked to firmly bite together whilst the clinician palpated the masseter muscle on the right side. A point of injection was determined (the most prominent part of the muscle, approximately 2 cm superior to the mandibular boarder) and marked with a felt pen. The point of injection was thereafter marked on a translucent paper where the participant’s nose, outline of the eye and jawline were marked. This was done in order to be able to identify the same infusion point for both infusions with the same substance.

The skin surface was cleaned with a disinfectant wipe and left to dry for at least 30 seconds before insertion of the sterile infusion needle (diameter 0.4 mm, length 19 mm). The needle was inserted into the relaxed muscle to a depth of approximately 17–18 mm. Each infusion was provided during 5 min from an infusion pump (infusion rate 30 mL/h total volume 2.5 mL; Harvard Infusion Pump 22, Harvard Apparatus, Great Britain)^19^.

The sterile buffered acidic saline solution (9 mg/ml, pH 5.2) was prepared by APL (Apotek Produktion & Laboratorier AB, Umeå, Sweden) and consisted of sodium dihydrogen phosphate dehydrate 21.37 mg/ml, disodium phosphate dihydrate 0.52 mg as well as sterile water. The control substance was a sterile isotonic saline solution consisting of monosodium chloride 9 mg/mL, pH 6 (Fresenius Kabi, Uppsala, Sweden).

### Chewing test

The participants were asked to chew two pieces of chewing gum (V6, Kraft Foods, Upplands Väsby, Sverige) for one minute. Pain intensity (NRS 0–10) and fatigue (Borg’s RPE 6–20) were recorded immediately after.

### Data analysis and statistics

For comparison of variables assessed repeatedly during day 1 and day 3 the recordings taken before (baseline), during and 5, 15, and 30 min after infusions were used. For comparisons between the different days the recordings taken at baseline day 1, directly after the infusion (AI) on day 1 and day 3 as well as on day 4 and 7 were used.

The PPT values were normalized to baseline, that is, the relative changes (%) were used in the statistical analyses. For the recordings that were done 5, 15, and 30 min after infusions, the baseline value from the same day was used, i.e. for day 3 baseline 2 was used. For the comparisons between days 1, 3, 4, and 7 the baseline at day 1 was used.

SigmaPlot software (version 14.0, Systat software Inc., San Jose, CA, USA) was used for all statistical analysis. The Shapiro-Wilk test for continuous variables was used to test the normal distribution. Since most of the variables were not normally distributed, non-parametric statistics were used for all variables, but the PPT that showed a normal distribution. Mean values and standard deviations (SD) were used for PPT while median (IQR) were used for the other variables for descriptive data.

Friedman repeated measures of analysis of variance on ranks (Friedman RM ANOVA) was used to analyze differences in pain intensity, fatigue, MUO as well as pain spread over time. When a significant difference was indicated, the Tukey test for multiple comparisons versus a control group (baseline) was used as a post hoc test. To compare differences between substances at the various time points Wilcoxon test was used with Bonferroni correction for multiple comparisons. As there were in total 22 comparisons made for these variables, the significance level of *P* < 0.0023 was set for this analysis.

Two-way repeated measures of analysis of variance (2-way RM ANOVA) with substance as the independent factor and time as the repeated factor was used to analyze changes of PPT. When the RM ANOVA indicated a significant difference, the Holm-Sidak test for multiple comparison versus a control group (baseline) was used as a post hoc test to test differences between substances and interactions at the different time point.

The significance level was set to p < 0.05 for all tests.

### Ethics approval and consent to participate

The project was approved by the Regional Ethical Review Board in Stockholm, Sweden, (Dnr. 2014/466-31/4) and followed the guidelines according to the Declaration of Helsinki as well as Good Clinical Practice. The participants received both written and oral information about the study and gave their written informed consent.
